# Pulmonary involvement in Gaucher disease

**DOI:** 10.1590/0100-3984.2016.0081

**Published:** 2017

**Authors:** Lucas de Pádua Gomes de Farias, Igor Gomes Padilha, Carla Jotta Justo dos Santos, Carol Pontes de Miranda Maranhão, Christiana Maia Nobre Rocha de Miranda

**Affiliations:** 1 Universidade Federal de Alagoas (UFAL), Maceió, AL, Brazil; 2 Clínica de Medicina Nuclear e Radiologia de Maceió (MedRadius), Maceió, AL, Brazil

*Dear Editor*,

A 33-month-old female patient was referred to the radiology department for evaluation of a two-week history of tachycardia syndrome, presenting without fever or general impairment. She was the daughter of consanguineous parents (first cousins) and had been diagnosed at 7 months of age with Gaucher disease (GD) type 2, on the basis of the evaluation of enzymatic activity. An initial investigation with conventional chest X-ray (Figure 1) revealed a bilateral reticulonodular interstitial pattern. Multidetector computed tomography (MDCT) revealed marked, diffuse thickening of the interlobular and intralobular septa, interspersed with areas of lesser involvement, accompanied by ground-glass opacity of the lung parenchyma, characterizing the crazy-paving pattern (Figure 2).

**Figure 1:**
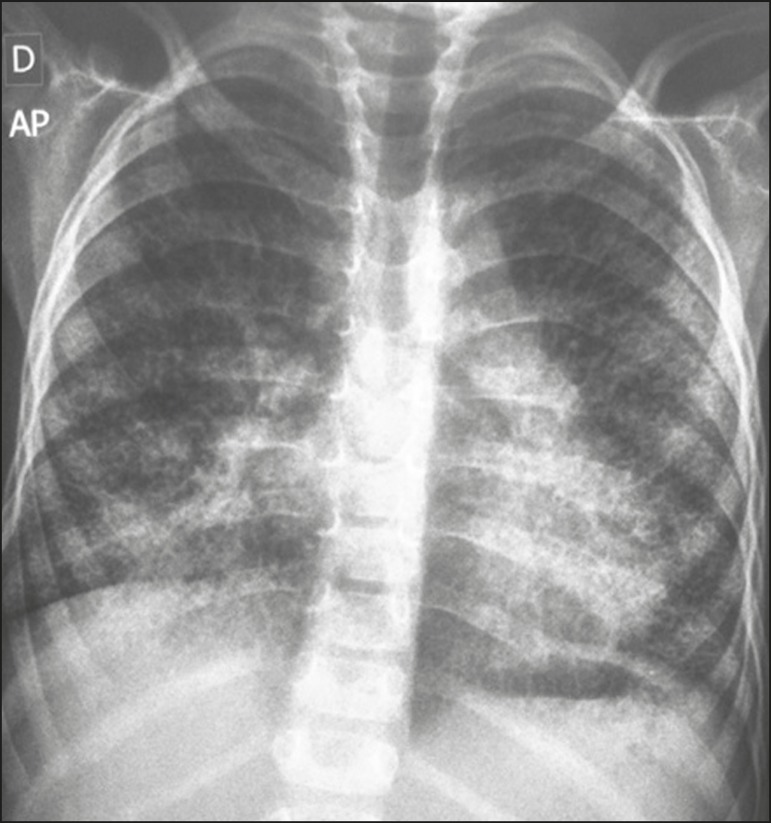
Anteroposterior chest X-ray showing a bilateral reticulonodular interstitial pattern that is more pronounced in the lower lobes.

**Figure 2:**
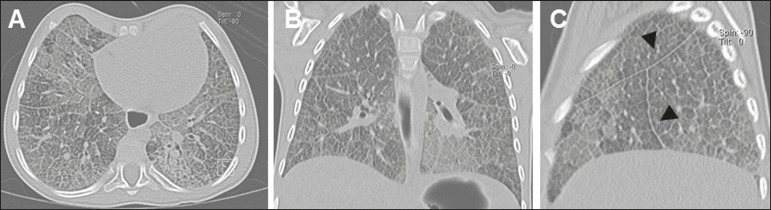
Axial (**A**), coronal (**B**), and sagittal (**C**) MDCT scans of the right lung showing diffuse, marked thickening of the interlobular and intralobular septa, accompanied by ground-glass opacity of the lung parenchyma, characteristic of the crazy-paving pattern. Note also the irregularity with the pleural surface and the thickening of the fissures (arrowheads).

GD has an autosomal recessive pattern of inheritance and corresponds to glucocerebrosidase deficiency, resulting in the accumulation of glucocerebrosides in macrophages of the reticuloendothelial system; macrophages that have thus been altered are referred to as Gaucher cells^(1-3)^. That accumulation mainly causes hyperplasia of the liver, spleen, and lymph nodes, hepatosplenomegaly being the principal characteristic of the disease. The lungs, skin, eyes, kidneys, and heart are rarely involved^(1-6)^. GD is the most prevalent lysosomal storage disease and is traditionally classified into three major phenotypes: type 1 (the chronic, non-neuropathic, adult type), which accounts for 99% of all cases and is characterized by a clinical profile with little clinical evidence; type II (the acute, neuropathic, infantile type), which usually results in death before the age of two years due to pneumonia and anoxia; and type III (the subacute, neuropathic, juvenile type), which has a heterogeneous course. Other less prevalent types are the perinatal-lethal and cardiovascular forms^(2-6)^.

Although pulmonary involvement is considered rare in GD, it has been frequently identified. However, there have been no epidemiological studies of the issue. In the literature, there is a lack of standardization of the radiological presentations of GD, due to the multifactorial involvement with multiple patterns of tissue infiltration by Gaucher cells^(4,6,7)^.

The imaging characteristics of GD correspond to several pathophysiological mechanisms. In addition to thickening of the interlobular and intralobular septa, patients with GD can present with alveolar opacities, capillary plugging by Gaucher cells, and interstitial opacities, with a predominance of lymphatic distribution, as well as respiratory infections^(4-8)^. Other alterations described include pulmonary fibrosis, a miliary pattern and involvement of the hilar or mediastinal lymph nodes, as well as a reduction in lung volume as a consequence of hepatosplenomegaly. Radiographic examinations can reveal an interstitial pattern and can show any changes in bone structures^(3-7)^.

The diffuse pulmonary involvement seen in patients with GD indicates that it is a systemic disease. MDCT is an important tool for the initial evaluation and follow-up of these patients, and lung biopsy can be dispensed with when the tomography reveals interstitial opacities in an appropriate clinical and epidemiological context^(6,7)^.

When there is no clinical suspicion of GD, a tomographic finding of the crazy-paving pattern makes the radiologic diagnosis difficult^(9)^. In such cases, the main differential diagnoses are alveolar proteinosis, pulmonary hemorrhage, pulmonary vasculitis, diffuse alveolar damage (acute respiratory distress syndrome), pulmonary edema, bronchioloalveolar carcinoma, Niemann-Pick disease, and radiation pneumonitis, as well as *Pneumocystis*, viral, lipoid, mycobacterial, interstitial, and eosinophilic pneumonia.

## References

[1] Beutler E (1991). Gaucher's disease. N Engl J Med.

[2] Pastores GM, Hughes DA, Adam MP, Ardinger HH, Pagon RA (1993). Gaucher disease. 2000 Jul 27 [Updated 2015 Feb
26]. GeneReviews©.

[3] Mendonça VF, Paula MTM, Fernandes C (2001). Manifestações esqueléticas da doença
de Gaucher. Radiol Bras.

[4] Wolson AH (1975). Pulmonary findings in Gaucher's disease. Am J Roentgenol Radium Ther Nucl Med.

[5] Kerem E, Elstein D, Abrahamov A (1996). Pulmonary function abnormalities in type I Gaucher
disease. Eur Respir J.

[6] Aydin K, Karabulut N, Demirkazik F (1997). Pulmonary involvement in adult Gaucher's disease high resolution
CT appearance. Br J Radiol.

[7] Amir G, Ron N (1999). Pulmonary pathology in Gaucher's disease. Hum Pathol.

[8] Yassa NA, Wilcox AG (1998). High-resolution CT pulmonary findings in adults with Gaucher's
disease. Clin Imaging.

[9] Müller CIS, D'Ippolito G, Rocha AJ (2010). Tórax.

